# Effects of virtual reality-based simulation education for nursing students: a systematic review and meta-analysis

**DOI:** 10.7586/jkbns.25.088

**Published:** 2026-02-27

**Authors:** Yean-Hee Jeong, Jihoo Her

**Affiliations:** College of Nursing, Gimcheon University, Gimcheon, Korea

**Keywords:** Students, nursing, Virtual reality, Simulation training, Systematic review, Meta-analysis

## Abstract

**Purpose:**

This study conducted a systematic analysis of the effects of virtual reality (VR)-based simulation education on nursing students’ learning outcomes.

**Methods:**

Studies involving undergraduate nursing students who received immersive or non-immersive VR–based simulation education, compared with conventional nursing education, were identified from Cochrane CENTRAL, EMBASE, PubMed, CINAHL, ACM Digital Library, NDSL, KISS, DBpia, and RISS, from database inception through February 28, 2025. The primary outcomes were nursing skill performance and knowledge, while the secondary outcomes were self-efficacy and confidence in nursing performance. Only randomized controlled trials were included. A total of 21 studies met the inclusion criteria and were synthesized using a random-effects meta-analysis. Effect sizes were calculated as standardized mean differences (SMDs) with 95% confidence intervals (CIs).

**Results:**

Across 21 randomized controlled trials, the meta-analysis showed that VR-based simulation education had no statistically significant effects on nursing skill performance (n = 759, SMD = 0.18; 95% CI: −0.47 to 0.82) or confidence in nursing performance (n = 1,157, SMD = 0.12; 95% CI: −0.20 to 0.43). In contrast, statistically significant positive effects were observed for knowledge (n = 1,009, SMD = 0.32; 95% CI: 0.08 to 0.57) and self-efficacy (n = 696, SMD = 0.46; 95% CI: 0.11 to 0.81).

**Conclusion:**

VR-based simulation education represents an effective educational approach for improving nursing students’ knowledge and self-efficacy. Future implementations should align the type and complexity of VR simulations with specific educational goals and content, while accounting for potential variation related to instructional design, learner characteristics, and outcome measures.

## INTRODUCTION

Nursing is a practice of caring that involves assessing and addressing human responses to actual or potential health problems [1]. Nurses are required to integrate knowledge, clinical skills, professionalism, and beliefs to provide autonomous and accountable nursing care, and such competencies that are developed through education [2,3]. Although nursing students prepare for their professional roles through theoretical education and clinical practice, the gap between theory and actual clinical settings has been reported to hinder their adaptation to clinical environments and professional performance [4]. Thus, it is essential to develop and implement practical training programs that provide direct experiences in environments similar to real clinical situations.

Simulation−based education is one strategy to address these educational needs, providing nursing students with safe and repetitive practice opportunities [5]. However, due to the high costs of equipment and facilities, limited scenarios, and constraints on time and space, it is difficult to provide sufficient opportunities for all learners [6,7]. Virtual reality (VR)-based simulation has gained attention as an alternative to overcome these limitations. By utilizing computer graphics, sensors, and head-mounted displays (HMDs) [6,8], VR can generate realistic virtual environments, enabling repeated learning and providing individualized feedback without time and place restrictions [9,10].

VR technology can be described as either immersive or non-immersive, depending on how strongly it engages the user. Immersive VR delivers a heightened sense of presence and interaction by using devices such as HMDs [11], whereas non-immersive VR allows users to interactively control virtual environments displayed on a monitor using a mouse or controller [8]. These various forms of VR technology have been applied to simulation-based education to replicate realistic clinical scenarios, facilitating learners’ problem-solving and decision−making skills [12].

VR-based simulations can also be classified by their educational focus into skill-based and situation-based types. Skill-based simulations emphasize the accurate performance of specific nursing procedures, such as nasogastric tube feeding, urinary catheterization, and intravenous injection. In contrast, situation-based simulations focus on non−technical skills, particularly situational awareness, and deal with topics such as sudden changes in a patient’s condition or disaster response [13]. This classification helps to identify how VR contributes to improving either procedural skills or complex situational coping abilities. It also provides an important basis for evaluating the effectiveness of VR in achieving different educational objectives and for developing optimized VR-based educational scenarios.

Recent studies examining the effects of VR-based simulation in nursing education on various outcomes, including nursing skills, knowledge, self−efficacy, and confidence in nursing performance, have reported inconsistent findings [14−16]. The discrepancy in these findings may stem from differences in study design, type of VR, educational content and methods, and the outcome variables measured. Such variations have limited the effective implementation and broader uptake of VR-based approaches in nursing education. In particular, although acquiring nursing skills and knowledge is a fundamental goal of nursing education, the influence exerted by VR-based simulation on these outcomes has been inconsistent, indicating the need for more practical and objective verification.

Therefore, this study aimed to systematically search and analyze studies on VR-based simulation in nursing education published in domestic and international journals, and to conduct a meta-analysis that comprehensively considered the type of VR simulation, educational content, and outcome variables. Through this, the overall effects of VR-based simulation in nursing education are explored, providing evidence to guide the design and development of future VR-based simulation programs.

## METHODS

The methodological approach for this review was informed by widely accepted Cochrane guidance for conducting systematic reviews, and the reporting adhered to the Preferred Reporting Items for Systematic Reviews and Meta-Analyses (PRISMA) guidelines [17,18]. In accordance with recommended practice, this review was also registered in PROSPERO (https://www.crd.york.ac.uk/PROSPERO/view/CRD420251029154).

### 1. Eligibility criteria

The main questions of this systematic review were organized based on the Participants, Interventions, Comparators, Outcomes, and Study design (PICOS) framework. Participants were undergraduate nursing students enrolled in domestic or international nursing programs. Intervention was a simulation-based nursing education program using either immersive or non-immersive VR. Comparison included nursing education programs based on one or more traditional strategies such as simulators, videos, lectures, or printed materials. Outcomes were nursing skill performance and knowledge as the primary outcomes, and self-efficacy and confidence in nursing performance as the secondary outcomes. Eligibility was restricted to studies conducted as randomized controlled trials (RCTs).

The inclusion criteria were studies written in Korean or English, with no restrictions on the type of VR device or duration of the intervention. Only studies that provided sufficient statistical information for calculating effect sizes (sample size, mean, and standard deviation) were included. One included study measured two different nursing skills within the same sample; each outcome was analyzed separately without adjusting the sample size. Another study involved two experimental groups and one control group; to prevent double-counting of the shared control, the control group sample size was evenly divided and entered into the analysis.

The exclusion criteria specified that studies were not eligible if they used interventions other than VR-based simulation education, lacked accessible full text, did not include a control group, targeted healthcare professionals, or included heterogeneous or mixed participant groups.

### 2. Information sources

The literature search was conducted without restrictions on the starting year and included studies published up to February 28, 2025. The databases searched were Cochrane CENTRAL, Excerpta Medica Database (EMBASE), PubMed, Cumulative Index to Nursing and Allied Health Literature (CINAHL), and the Association for Computing Machinery (ACM) Digital Library, as well as the Korean databases National Digital Science Library (NDSL), Korean Studies Information Service System (KISS), DBpia, and Research Information Sharing Service (RISS). To enhance the sensitivity for identifying gray literature, a manual search was also conducted using Google Scholar.

### 3. Search strategy

The search strategy was developed based on the PICOS framework. After conducting preliminary searches and reviewing abstracts and indexing terms, comprehensive search terms were selected. Each database was queried using both its standardized indexing terms and additional free-text keywords. Specifically, MeSH terms were used for PubMed and CENTRAL, CINAHL Headings were used for CINAHL, and Emtree terms were used for EMBASE. Synonyms and alternative expressions were also identified, and Boolean operators such as AND, OR, and truncation symbols (*) have been applied to increase the sensitivity of the search. For Korean databases, major Korean keywords were combined and searched accordingly.

For nursing students in the PubMed database, the search terms included “Students, Nursing [MeSH Terms]” and “Nursing Student*”. For VR-based simulation, the search terms included “virtual reality [MeSH Terms]”, “virtual realit*”, “Virtual Simulat*”, “Virtual Learning Platform*”, “Head Up Display*”, “Head Mounted Display*”, “virtual education*”, “virtual patient*”, and “virtual game*”. Following the guidance of the Cochrane Handbook, which emphasizes maximizing search sensitivity, broad and inclusive search elements were employed (Appendix 1).

### 4. Selection process

**Figure 1.**
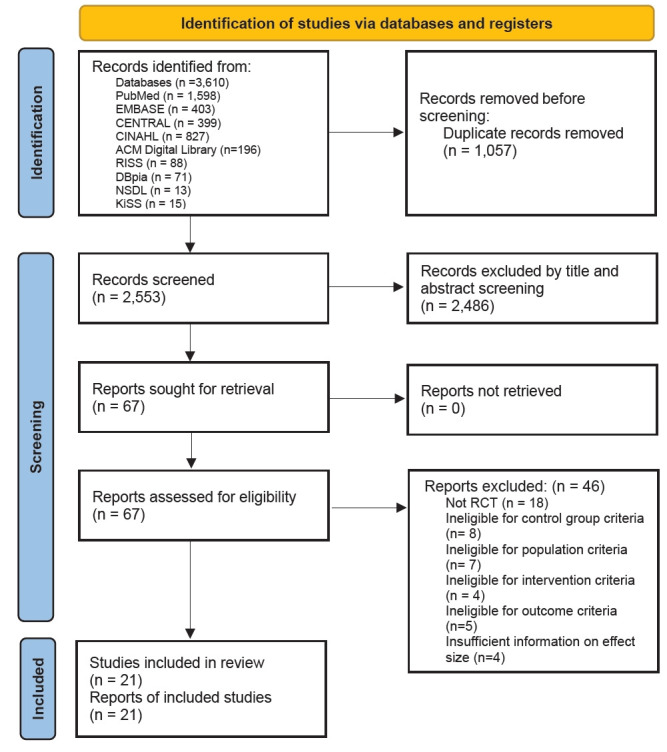
Flow diagram of study selection.

All retrieved records were screened independently by two reviewers. Following the removal of duplicate records, titles and abstracts were reviewed to assess eligibility, followed by full-text evaluation. Discrepancies between reviewers were resolved through discussion, and when agreement could not be reached, a third reviewer adjudicated the decision. Reasons for exclusion at the full-text review stage were documented. The study selection process is summarized in the PRISMA flow diagram (Figure 1).

### 5. Data collection process

All records retrieved from the database were processed using EndNote 20 for reference management. Duplicate studies were automatically identified using the “find duplicates” function, which compared titles, authors, publication years, and DOIs, and were subsequently verified and removed through manual review. The studies that met the final inclusion criteria were transferred to Microsoft Excel, version 16.0 (Microsoft Corporation, Redmond, WA, USA) for data management.

### 6. Data items

Data were extracted independently by two reviewers using a standardized form. The extracted data included the first author, country, publication year, study design, sample size, intervention characteristics, outcome measures, and main results. For meta-analysis, quantitative data required to calculate effect sizes, including group-specific sample sizes and post-intervention means and standard deviations, were also extracted. When information was missing or unclear, assumptions were made based on the available data, and discrepancies were resolved through discussion.

### 7. Study risk of bias assessment

The risk of bias of the included studies was assessed using the Cochrane Risk of Bias 2 (RoB 2) tool. This instrument evaluates five domains: bias arising from the randomization process, bias due to deviations from intended interventions, bias due to missing outcome data, bias in outcome measurement, and bias in the selection of reported results. Each domain was rated as “low risk,” “some concerns,” or “high risk,” according to the RoB 2 guidance [19]. Two reviewers independently assessed the risk of bias for each study. Disagreements were resolved through discussion or consultation with a third reviewer.

### 8. Effect measures

For continuous outcomes, effect sizes were calculated as standardized mean differences using Hedges’ g, estimated under a random-effects model based on the DerSimonian and Laird method, with corresponding 95% confidence intervals (CIs). Hedges’ g was selected because it provides a bias-corrected estimate of effect size and is more appropriate than Cohen’s d when included studies have relatively small sample sizes [17,20]. Outcome measures were oriented so that higher scores consistently indicated more favorable results.

### 9. Synthesis methods

Studies were grouped for each synthesis based on similarities in intervention characteristics, outcome measures, and comparison groups, as prespecified in the review protocol. Eligible studies were tabulated and compared against the planned synthesis groups to determine their inclusion in each meta-analysis.

Data extraction and analysis followed Cochrane guidelines. When necessary, summary statistics were converted into a common metric to enable quantitative synthesis. The results of individual studies and pooled estimates were visually presented using forest plots [21,22].

Meta-analyses were conducted using Review Manager version 5.4.1 (RevMan, The Nordic Cochrane Centre, Copenhagen, Denmark). Given the heterogeneity across studies, a random-effects model was applied. Statistical heterogeneity was assessed using visual inspection of forest plots and the Higgins I^2^ statistic, with I^2^ values greater than 50% or a *p* value < .05 in Cochran’s Q test indicating substantial heterogeneity. When substantial heterogeneity was identified, prespecified subgroup analyses were conducted to explore potential sources of heterogeneity based on study characteristics. Meta-regression analysis was not performed due to the limited number of studies included in each outcome [22–24].

In addition, a network meta-analysis was conducted for selected outcomes to compare the relative effects of different VR intervention configurations by simultaneously integrating direct and indirect evidence. The network meta-analysis was performed using a frequentist random−effects model with the netmeta package in R version 4.5.2 (R Foundation for Statistical Computing, Vienna, Austria), and effect estimates were expressed as standardized mean differences with 95% CIs.

### 10. Reporting bias assessment

Publication bias was assessed by visual inspection of funnel plots and Egger’s regression test using a random effects model implemented in R version 4.5.2 (R Foundation for Statistical Computing, Vienna, Austria) with the metafor package. Funnel plot symmetry was interpreted as indicating a lower likelihood of publication bias, whereas asymmetry suggested potential reporting bias [21,22,24]. When funnel plot asymmetry was suggested, the trim−and−fill method was applied to estimate the number of potentially missing studies and to examine the robustness of the pooled effect size after adjustment.

## RESULTS

### 1. Study selection

The selection of studies included in this analysis was conducted in accordance with the PRISMA guidelines, following three sequential phases: identification, screening, and inclusion. The detailed process is presented in Figure 1.

### 2. Characteristics of the included studies

The general characteristics of the 21 studies included in the systematic review, the characteristics of the interventions, and the outcomes regarding the effects of VR-based simulation in nursing education are summarized in Table 1.

Among the 21 studies included in this review, 19 were published after 2019, and 15 were conducted in Asian countries. VR was classified according to the level of learner interaction, with immersive VR defined as systems enabling active interaction with the virtual environment through the use of HMDs with controllers. In contrast, VR applications with limited interaction, including desktop or mobile-based systems or HMD-based experiences restricted to passive video viewing or VR document-based content, were classified as non-immersive VR. Based on these criteria, eight studies were classified as immersive VR and thirteen as non-immersive VR.

In terms of educational focus, 12 studies were skill-based, targeting core nursing procedures such as nasogastric tube feeding, urinary catheterization, and intravenous injection. The remaining 9 studies were situation-focused, addressing complex clinical scenarios that required integrated competencies, including fetal development, emergency response, and disaster management.

### 3. Risk of bias in studies

Of the 21 included studies, 8 (38%) were rated as low risk, 11 (52%) as having some concerns, and 2 (10%) as high risk. Overall, concerns were most frequently identified in the domains related to the randomization process and deviations from intended interventions (Appendix 2).

### 4. Meta-analysis of virtual reality–based simulation interventions

To evaluate the effects of VR-based simulation on nursing students, this study calculated effect sizes from 21 studies that reported sample size, mean, and standard deviation. The primary outcomes analyzed included nursing skill performance, knowledge level, self-efficacy, and confidence in nursing performance (Appendix 3).

Subgroup analyses were performed according to study characteristics. Specifically, VR was classified as immersive or non-immersive; control group education was categorized as simulator-based or non-simulator-based according to the use of simulators; and educational topics were classified as skill-oriented or scenario-oriented, with skill-oriented education focusing on the performance of core basic nursing skills and scenario-oriented education emphasizing the integrated application of competencies in complex clinical situations.

#### 1) Effect size of virtual reality–based simulation on nursing skill performance

Among the 21 included studies, 10 (Study ID: A1, A5, A7, A8, A10, A11, A15, A16, A17, and A20) assessed outcomes related to nursing skill performance. The pooled estimate showed an effect size of 0.18 (95% CI: −0.47 to 0.82; z = 0.53, *p* = .596), indicating that no statistically significant effect was identified.

In the subgroup analysis based on VR modality, neither immersive VR nor non-immersive VR showed a statistically significant effect on nursing skill performance. Similarly, no statistically significant effects were observed according to control group type, regardless of whether simulator-based or non-simulator-based education was used.

**Figure 2.**
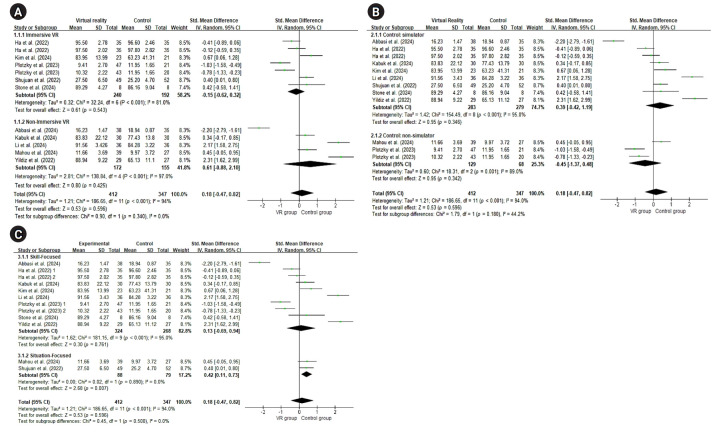
Forest plot of the effect of virtual reality-based simulation education on nursing skill performance. (A) Immersiveness of intervention (immersive vs. non-immersive). (B) Control group (simulator vs. non-simulator). (C) Contents type (skill focused vs. situation focused). SD = Standard deviation; CI = Confidence interval; VR = Virtual reality.

Regarding the nature of educational content, skill-focused interventions did not show a statistically significant effect on nursing skill performance. In contrast, situation-focused interventions yielded a statistically significant effect (effect size = 0.42; 95% CI: 0.11 to 0.73; z = 2.68; *p* = .007). However, the difference between skill-focused and situation-focused interventions was not statistically significant (*p* = .500) (Figure 2).

In addition, a network meta-analysis was conducted to compare multiple VR-based educational configurations combining immersion level (immersive vs. non-immersive), simulation focus (skill-based vs. situation-based), and comparator type (simulator-based vs. non-simulator-based education). The results showed that none of the VR-based educational configurations demonstrated a statistically significant advantage over simulator-based education in improving nursing skill performance (Appendix 3).

#### 2) Effect size of virtual reality-based simulation on knowledge

Among the 21 included studies, 14 (Study ID: A1, A2, A3, A4, A6, A7, A8, A9, A11, A12, A13, A15, A17, and A19) assessed outcomes related to knowledge acquisition. The pooled analysis indicated an effect size of 0.32 (95% CI: 0.08 to 0.57; z = 2.59, *p* = .010), demonstrating a statistically significant improvement in knowledge levels.

When comparing VR modalities, non-immersive VR showed a significant beneficial effect, with an effect size of 0.43 (95% CI: 0.08 to 0.79; z = 2.38, *p* = .017), whereas immersive VR did not show a statistically significant effect. The contrast between non-immersive and immersive VR was not statistically significant.

In the subgroup comparison based on control type, studies using simulator-based controls did not show a statistically significant effect. In contrast, studies employing non-simulator controls demonstrated a significant positive effect, with an effect size of 0.34 (95% CI: 0.06 to 0.61; z = 2.40, *p* = .016). However, the comparison between the two control conditions did not reveal a statistically significant difference.

**Figure 3.**
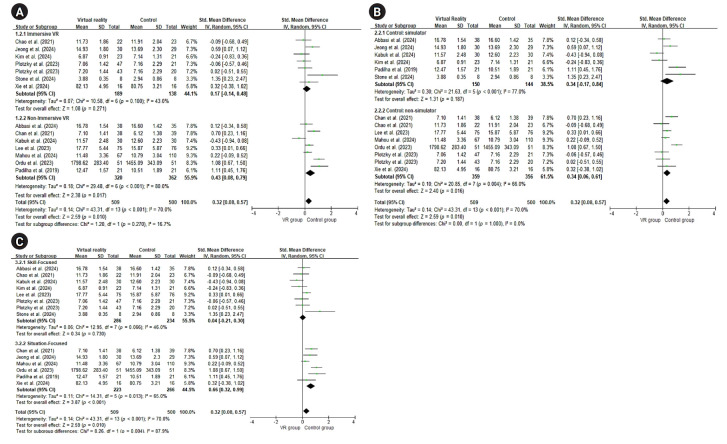
Forest plot ofj the effect of virtual reality-based simulation on knowledge. (A) Immersiveness of Intervention (immersive vs. non-immersive). (B) Control group (simulator vs. non-simulator). (C) Contents type (skill focused vs. situation focused). SD = Standard deviation; CI = Confidence interval; VR = Virtual reality.

When categorized by educational content, situation-based interventions produced a significant effect size of 0.66 (95% CI: 0.32 to 0.99; z = 3.87, *p* < .001). Skill-based interventions did not show a statistically significant improvement. The contrast between these two types of educational content was statistically significant (*p* = .004) (Figure 3).

To further compare the relative effectiveness of different VR-based educational configurations, a network meta-analysis using a random-effects model was conducted. The results indicated that both immersive VR-based situation-focused education (standardized mean differences [SMD] = 1.02, 95% CI: 0.26 to 1.78, *p* = .009) and non-immersive VR-based situation-focused education (SMD = 0.71, 95% CI: 0.08 to 1.35, *p* = .028) demonstrated significantly greater improvements in knowledge outcomes compared with simulator-based education. In contrast, neither immersive nor non-immersive skill-focused VR interventions showed a statistically significant advantage over simulator-based education (Appendix 4).

#### 3) Effect size of virtual reality–based simulation on self-efficacy

A total of seven studies examined outcomes related to self-efficacy. The pooled analysis showed a statistically significant effect, with an effect size of 0.46 (95% CI: 0.11 to 0.81; z = 2.57, *p* = .008), indicating that VR-based simulation significantly improved self-efficacy compared with control conditions (Appendix 5).

#### 4) Effect size of virtual reality-based simulation on confidence in nursing performance

Twelve studies assessed confidence in nursing performance. The overall analysis showed no statistically significant effect of VR-based simulation, with an effect size of 0.12 (95% CI: −0.20 to 0.43; z = 0.74, *p* = .460), indicating no meaningful improvement in confidence compared with control conditions (Appendix 6).

### 5. Publication bias

For skill performance outcomes (k = 12), Egger’s regression test did not indicate significant funnel plot asymmetry (z = 0.65, *p* = .513). Trim-and-fill analysis suggested no missing studies, and the adjusted pooled effect size remained non-significant (SMD = 0.18, 95% CI: −0.53 to 0.89, *p* = .625), suggesting that the results were not materially influenced by publication bias.

For knowledge outcomes (k = 14), Egger’s regression test did not indicate significant funnel plot asymmetry (z = 1.64, *p* = .101). Trim-and-fill analysis suggested one potentially missing study, and the adjusted pooled effect size was slightly attenuated but remained statistically significant (SMD = 0.33, 95% CI: 0.01 to 0.65, *p* = .042), indicating robustness of the findings to potential publication bias.

For confidence in nursing performance outcomes (k = 13), Egger’s regression test did not indicate significant funnel plot asymmetry (z = −0.62, *p* = .536). Trim-and-fill analysis suggested no missing studies, and the adjusted pooled effect size remained non−significant (SMD = 0.12, 95% CI: −0.21 to 0.45, *p* = .486), suggesting that the findings were not materially influenced by publication bias.

**Figure 4.**
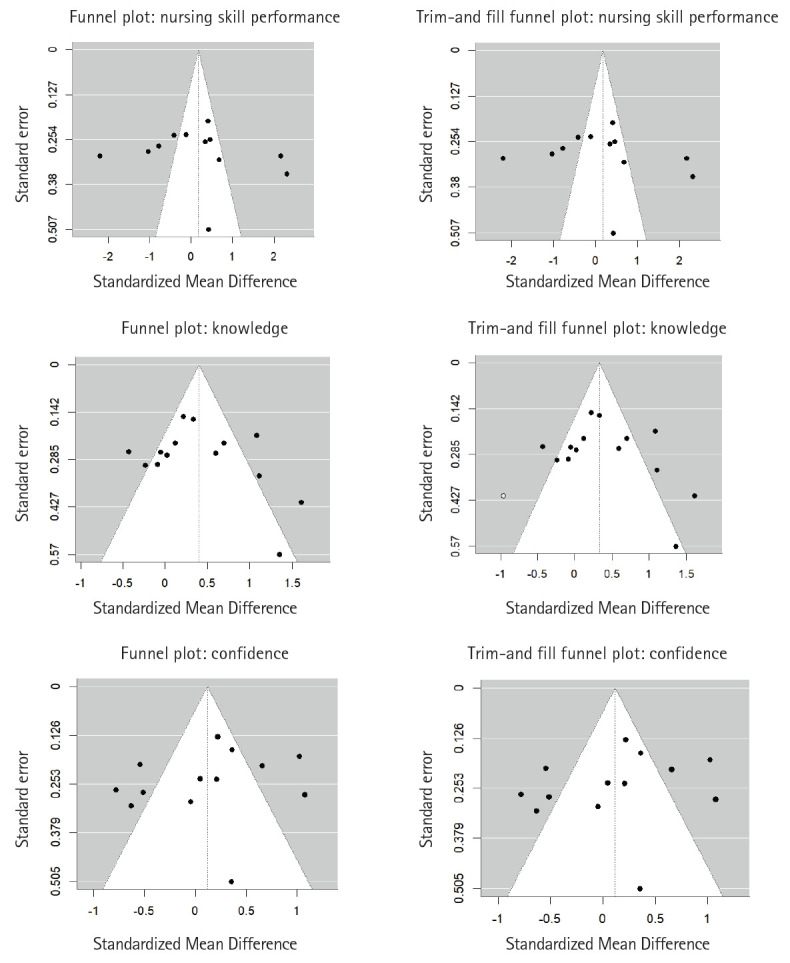
Funnel plot of comparison.

Publication bias was not formally assessed for self-efficacy outcomes due to the limited number of included studies. Funnel plots and trim-and-fill funnel plots for skill performance, knowledge, and confidence in nursing performance outcomes are presented in Figure 4.

## DISCUSSION

This study was conducted to examine the effects of VR-based simulation education among nursing students. Based on this, the trends of VR-based simulation studies in nursing education were analyzed. Research on VR-based simulation in nursing education has shown a gradual increase since the first study published in 2016, reaching 10 studies in 2024. This increase can be interpreted as the result of multiple factors acting together, including the advancement and accessibility of VR technology, the growing demand for non-face-to-face education during the coronavirus disease 2019 pandemic, and the need for innovation in nursing education [25].

By study type, non-immersive VR studies (n = 13) outnumbered immersive VR studies (n = 8). Non-immersive studies have steadily increased since 2016, whereas immersive studies have rapidly expanded since 2022. This trend suggests that, while non-immersive VR was initially preferred due to its higher accessibility, the recent development of immersive technologies and the wider distribution of related equipment have led to a sharp increase in immersive VR studies [26]. Analysis by topic type showed that, among skill-based studies, simulator-controlled trials were predominant (n= 9), whereas non-simulator-based studies were limited (n = 3). This reflects a research tendency to compare the effectiveness of VR with conventional simulator-based education. Such control group compositions have an important influence on the interpretation of effect sizes [27]. In contrast, among situation-based studies, non-simulator-based control groups were more common, indicating that VR has often been compared with traditional lecture- or discussion-based education. Therefore, VR has been applied as a complementary tool to simulator-based education in skill-based topics and as a new educational approach to reproduce complex clinical situations in situation-based topics.

According to the meta-analysis results, VR-based simulation showed limited effects on nursing skill performance, whereas it demonstrated beneficial effects on knowledge acquisition. These patterns were further supported by the network meta-analysis, which showed no statistically significant differences among various VR configurations for nursing skill performance, whereas situation-focused VR interventions demonstrated a consistent advantage over simulator-based education for knowledge outcomes.

This suggests that while VR has strengths in promoting cognitive engagement and knowledge internalization [28−30], it may have limitations in transferring tactile sensations, fine motor skills, and muscle memory required for actual skill performance [31].

VR-based simulation offers educational benefits by enabling learners to transform abstract concepts into concrete understanding through realistic visual and auditory experiences [32], and by facilitating problem-solving through repeated decision-making practice in a virtual environment [33,34] However, its current technological constraints limit the accurate reproduction of fine sensory feedback essential for hands-on clinical skills [31].

Among the included studies, some reported significant improvements in both the experimental and control groups after the intervention [35,36], suggesting that it may have been difficult to isolate the independent effect of VR alone [37]. In fact, several meta-analyses that included studies without additional educational interventions in the control group reported that VR was effective in improving skill performance [28,30]. This implies that the learning effect may have resulted more from the structured educational intervention itself than from the inherent effect of VR simulation.

In the subgroup analysis, a statistically significant pooled estimate for nursing skill performance was observed in situation-based VR interventions. However, this finding should be interpreted with caution. The difference between situation-based and skill-based interventions did not reach statistical significance, and when multiple intervention characteristics-including immersion level, educational focus, and comparator type-were simultaneously considered in the network meta-analysis, no VR-based configuration demonstrated a statistically significant advantage over simulator-based education.

These findings suggest that the apparent benefit observed in situation−based topics may not represent a robust or independent effect of VR itself, but rather reflects sensitivity to study design features and comparator characteristics. In particular, the frequent use of non-simulator-based control groups in situation-based studies, in contrast to simulator-based controls commonly used in skill-based studies, may have contributed to the observed variability in effect estimates. Therefore, while situation-based VR interventions may offer contextual advantages for learning, current evidence does not support a definitive conclusion that they are superior for improving nursing skill performance.

Although moderate to substantial heterogeneity was observed for several outcomes, the overall direction of the pooled effects remained consistent after adjustment using the trim-and-fill method. In particular, the knowledge outcome retained its statistical significance following adjustment, while the non-significant findings for nursing skill performance and confidence remained unchanged. These findings suggest that, despite between-study variability, the main conclusions of this meta-analysis are relatively robust to potential publication bias. However, this does not imply that heterogeneity was fully resolved, and the results should still be interpreted with consideration of contextual differences across studies.

This study explored various effects of VR-based simulation on nursing education beyond nursing skill performance and knowledge acquisition. The results showed a positive effect on self-efficacy, whereas no statistically significant difference was observed in nursing performance confidence. These findings contribute to a more comprehensive understanding of the educational utility of VR simulation. First, VR-based simulation education significantly improved self-efficacy, particularly in immersive environments and situation-based topics. This type of simulation enhances learning engagement by stimulating learners’ senses and maximizing a sense of realism as if they were experiencing actual clinical situations [38,39]. The increased sense of realism and immersion strengthens clinical decision-making and coping strategies [40], thereby contributing to the improvement of self-efficacy. In addition, subgroup analyses by control group type showed that VR-based simulation was more effective in enhancing self-efficacy than simulator-based education. This may be because VR can reproduce a wider range of clinical scenarios and enhance interactivity with learners, thereby expanding their overall learning experience [41].

In contrast, VR-based simulation did not show a statistically significant difference in improving nursing performance confidence. A similar trend was observed in the subgroup analysis, which is consistent with the earlier finding that there was no significant difference in nursing skill performance. Some of the studies included in this review modified the performance checklists into self-reported confidence tools for measuring nursing skills [42,43], and such measurement methods are closely related to actual performance ability. Therefore, considering that the improvement in skill performance was limited in this study, the absence of a significant change in performance confidence can be interpreted as a consistent finding. Nevertheless, a significant improvement in nursing performance confidence was observed in the situation-based topics. This suggests that the improvement in confidence through VR simulation is not primarily derived from the enhancement of technical nursing skills, but rather from the direct experience of a learning environment that reproduces complex clinical situations and requires integrative judgment. Nursing students may gain greater educational benefits by practicing independent judgment and decision−making as learners in situations that are difficult to experience directly before entering clinical practice. Therefore, when designing VR simulations to enhance nursing performance confidence, it is important to focus on developing problem-solving and coping abilities applicable to real clinical situations.

This study systematically analyzed the effects of VR-based simulation education for nursing students and provided an integrated perspective on both the educational potential and limitations of VR. Only published RCTs were included in the analysis to ensure objectivity and reliability. In addition, various subgroup analyses-such as those based on the type of immersion and control group were conducted to comprehensively explore the factors influencing the effects. During this process, some findings that contrasted with previous studies were identified, highlighting the limitations of VR and guiding directions for improvement. Furthermore, by providing quantitative evidence for various learning outcomes including knowledge, self-efficacy, and nursing performance confidence, this study provides evidence to inform for future development of tailored VR designs and scenarios.

The limitations of this study are as follows. First, although a comprehensive search strategy was applied, the review process may have been subject to potential selection bias due to the exclusion of non-English publications and unpublished studies. In addition, the number of studies included in some subgroup analyses was limited, which may have introduced uncertainty in estimating the effect sizes.

Second, the included studies showed a high degree of heterogeneity, which may have resulted from differences in intervention types, number of sessions, exposure duration, composition of control groups, and measurement tools. This heterogeneity suggests that contextual factors should be carefully considered when interpreting the pooled results.

Considering these limitations, future research should aim to standardize comparison groups, clarify the dose–response relationship of topics, immersion levels, and exposure intensities, ensure consistent use of objective outcome indicators, and further minimize potential publication bias through strengthened registration and reporting systems.

## CONCLUSION

The meta-analysis of VR-based simulation in nursing education suggested beneficial effects in several domains compared with traditional education. Although no consistent significant differences were found in nursing skill performance or performance confidence, meaningful improvements were observed in knowledge and self-efficacy. In particular, situation-based topics showed positive outcomes in multiple aspects, and immersive VR tended to promote learner engagement and immersion. For educational applications, a blended strategy that appropriately combines VR and simulator-based training according to learning objectives and content is recommended. Given the variability across studies, developing situation-based content using immersive technologies may further enhance clinical decision−making and problem-solving competencies, whereas simulator-based education may remain more effective when the goal is to directly improve psychomotor performance.

## Figures and Tables

**Table 1.** Descriptive Summary of Studies Included in the Systematic Review

Study	Author (year)	VR type	Simulation type	Number of sessions / session duration (min)	Sample size (n)		Main outcome measure	Study results	Group comparison (E vs. C)
	Country				Experimental group	Control group		M ± SD	
A1	Abbasi (2024)	n-IVR	Skill-focused	1/NA	DVR (38)	Simulator (35)	1) Skill performance	1) E: 16.23 ± 1.47	1) E < C
	Iran		-CPR					C: 18.94 ± 0.87	
							2) Knowledge	2) E: 16.78 ± 1.54	2) No difference
								C: 16.60 ± 1.42	
A2	Chan (2021)	n-IVR	Situation-focused	Once a week/NA	HMD (VR Docs) (38)	Printed material (39)	1) Knowledge	1) E:7.10 ± 1.41	1) C < E
	Taiwan		-Chemotherapy administration					C: 6.12 ± 1.38	
A3	Chao (2021)	n-IVR	Skill-focused	1/10-20	HMD (VR Docs) (22)	Video (23)	1) Knowledge	1) E:11.73 ± 1.86	1) No difference
	Taiwan		-Nasogastric tube feeding					C:11.91 ± 2.04	
							2) Confidence	2) E:15.41 ± 4.11	2) No difference
								C:15.61 ± 3.86	
A4	Cobbett (2016)	n-IVR	Situation-focused	2/45	DVR (27)	Simulator (28)	1) Knowledge	1)① E:4.12 ± 1.54	1)① No difference
	Canada		-Maternal-newborn nursing course				① Preeclampsia	C:4.80 ± 1.19	② No difference
							② GBS	② E:6.40 ± 1.73	
								C:6.82 ± 1.25	
							2) Confidence	2) E:104.89 ± 17.52	2) No difference
								C:115.25 ± 21.95	
A5	Ha (2022)	IVR	Skill-focused	1/120	HMD, Controller (35)	Simulator (35)	1) Performance	1)① E:95.50±2.78	1)① No difference
	Korea		-Foley catheterization				① Foley	C:96.60±2.46	② No difference
			-Tracheal tube care				② T-tube	② E:97.50±2.02	
								C:97.80±2.82	
							2) Confidence	2)① E:193.30±20.33	2)① No difference
							① Foley	C:189.30±18.03	② No difference
							② T-tube	② E:126.20±13.09	
								C:125.60±12.57	
							3) Self-efficacy	3) E:43.60±6.22	3) No difference
								C:42.50±5.11	
A6	Jeong (2024)	IVR	Situation-focused	1/120	HMD, Controller (30)	Simulator (29)	1) Knowledge	1) E:14.93 ± 1.80	1) C < E
	Korea		-Normal vaginal delivery care					C:13.69 ± 2.30	
							2) Self-efficacy	2) E:79.10 ± 6.43	2) C < E
								C:74.34 ± 8.40	
							3) Confidence	3) E:35.70 ± 3.05	3) C < E
								C:30.45 ± 6.13	
A7	Kabuk (2024)	n-IVR	Skill-focused	1/45	DVR (30)	Simulator (30)	1) Skill performance	1) E:83.83 ± 22.12	1) No difference
	Turkey		-CPR					C:77.43 ± 13.79	
							2) Knowledge	2) E:11.57 ± 2.48	2) No difference
								C:12.60 ± 2.23	
							3) Confidence	3) E:28.66 ± 6.95	3) C < E
								C:32.86 ± 2.87	
		
A8	Kim (2024)	IVR	Skill-focused	E:3/30	HMD, Controller (23)	Simulator (21)	1) Skill performance	1) E:83.95 ± 13.99	1) C < E
	Korea		-CPR	C:1/240				C:63.23 ± 41.31	
							2) Knowledge	2) E:6.87 ± 0.91	2) No difference
								C:7.14 ± 1.31	
							3) Self-efficacy	3) E:47.47 ± 5.19	3) No difference
								C:47.28 ± 4.44	
							4) Confidence	4) E:74.78 ± 8.39	4) No difference
								C:79.76 ± 6.93	
A9	Lee (2023)	n-IVR	Skill-focused	1/120	HMD (VR videos) (75)	Online lecture (76)	1) Knowledge	1) E:17.77 ± 5.44	1) C < E
	Hong Kong		-Blood transfusion					C:15.87 ± 5.87	
							2) Self-efficacy	2) E:29.83 ± 4.20	2) No difference
								C:29.32 ± 4.42	
							3) Confidence	3) E:24.69 ± 6.13	3) C < E
								C:22.75 ± 4.44	
A10	Li (2024)	n-IVR	Skill-focused	1/120	DVR (36)	Simulator (36)	1) skill performance	1) E:91.56 ± 3.43	1) C < E
	China		-CPR					C:84.28 ± 3.22	
A11	Mahou (2024)	n-IVR	Situation-focused	1/120	DVR	Printed material	1) Skill performance	1) E:11.66 ± 3.69	1) No difference
	Morocco		-Medication administration and dosage calculation		1) (39)	1) (27)		C:9.97 ± 3.72	
					2) (67)	2) (110)	2) Knowledge	2) E:11.48 ± 3.36	2) No difference
					3) (140)	3) (104)		C:10.79 ± 3.04	
							3) Confidence	3) E:33.0 ± 6.51	3) C < E
								C:31.6 ± 6.21	
A12	Ordu (2023)	n-IVR	Situation-focused	1/60	DVR (51)	Lecture (51)	1) Knowledge	1) E:1798.62±283.40	1) C < E
	Turkey		-Nursing process					C:1455.09±343.09	
A13	Padilha (2019)	n-IVR	Situation-focused	1/45	DVR (21)	Simulator (21)	1) Knowledge	1) E:12.47 ± 1.57	1)_C < E
	Portugal		-Respiratory, cardiac, and urinary systems					C:10.51 ± 1.89	
							2) Self-efficacy	2) E:30.38 ± 4.57	2) No difference
								C: 30.14 ± 4.29	
A14	Pauli (2024)	n-IVR	Skill-focused	1/20-30	DVR (56)	Simulator (46)	1) Knowledge and skills	1) E:70.27 ± 13.65	1) E < C
	USA		-Airway management					C:77.61 ± 11.18	
							2) Confidence	2) E:29.20 ± 5.15	2) E < C
								C:31.83 ± 4.33	
A15	Plotzky (2023)	IVR	Skill-focused	2026-01-20	HMD (high/low), Controller (47/43)	Video (41)	1) Skill performance	1)① E: 9.41 ± 2.70	1)① E < C
	Germany	(high/low)	-Endotracheal suctioning skill				① VR _high_	C:11.95 ± 1.65	② E < C
							② VR _low_	② E: 10.32 ± 2.22	
								C:11.95 ± 1.65	
							2) Knowledge	2)① E:7.06 ± 1.42	2) No difference
							① VR _high_	C:7.16 ± 2.29	
							② VR _low_	② E:7.20 ± 1.44	
								C:7.16 ± 2.29	
A16	Shujuan (2022)	IVR	Situation-focused	Once a week	HMD, Controller (49)	Simulator (52)	1) Performance	1) E:27.50 ± 6.50	1) C < E
	China		-Disaster response	/10-25				C:25.20 ± 4.70	
							2) Confidence	2) E:6.40 ± 1.20	2) C < E
								C:5.50 ± 1.50	
A17	Stone (2024)	IVR	Skill-focused	1/NA	HMD, Controller (8)	Simulator (8)	1) Performance	1) E:89.29 ± 4.27	1) No difference
	USA		-Perineal care					C:86.16 ± 9.04	
							2) Knowledge	2) E:3.88 ± 0.35	2) C < E
								C:2.94 ± 0.86	
							3) Confidence	3) E:4.41 ± 0.26	3) No difference
								C:4.31 ± 0.27	
A18	Tamilselvi (2024)	n-IVR	Situation-focused	1/4 hrs	DVR (70)	Printed Material (70)	1) Self-efficacy	1) E:41.06 ± 8.29	1) C < E
	India		-MI management					C:31.04 ± 9.42	
							2) Confidence	2) E:41.40 ± 9.20	2) C < E
								C:32.10 ± 8.88	
A19	Xie (2024)	IVR	Situation-focused	Two weeks/NA	HMD, Controller (16)	Video (16)	1) Knowledge	1) E:82.13 ± 4.95	1) No difference
	China		-Care of common diseases of older adults					C:80.75 ± 3.21	
A20	Yildiz (2022)	n-IVR	Skill-focused	10 hrs/15-17	DVR (29)	Simulator (27)	1) Skill performance	1) E:88.94 ± 9.22	1) C < E
	Turkey		-Intravenous catheterization					C:65.13 ± 11.12	
A21	Zahran (2025)	IVR	Skill-focused	E:8-9/15-20	HMD, Controller (95)	Simulator (95)	1) Self-efficacy	2) E:4.24 ± 0.28	2) C < E
	Palestine		-Airway management	C: 2/160-210				C:4.05 ± 0.14	

M = Mean; SD = Standard deviation; NS = Not significant; NA = Not available; E = Experimental group; C = Control group; VR = Virtual reality; IVR = Immersive virtual reality; n-IVR = non-Immersive virtual reality; HMD = Head-mounted display; DVR = Desktop virtual reality; CPR = Cardiopulmonary resuscitation; GBS = Group B streptococcus; MI = Myocardial infarction; T-tube = Tracheal tube.

## Data Availability

The data that support the findings of this study are available from the corresponding author upon reasonable request.
