# Supplementary material for: Effects of virtual reality-based simulation education for nursing students: a systematic review and meta-analysis
Source: J Korean Biol Nurs Sci. 2026 Feb 27;28(1):43–65. doi: 10.7586/jkbns.25.088 (PMC13268350; doi:10.7586/jkbns.25.088)
Supplement: Supplementary file 1 [file jkbns-25-088-Appendix-1.pdf]

Appendix 1. Searching Strategy

No.	Searching term	No.	Searching term
1	Nursing Student*	8	Head Up Display*
2	Students, Nursing [MeSH Terms]	9	Head Mounted Display*
3	1 OR 2	10	virtual education*
4	virtual reality [MeSH Terms]	11	virtual patient*
5	virtual realit*	12	virtual game*
6	Virtual Simulat*	13	4 OR 5 OR 6 OR 7 OR 8 OR 9 OR 10 OR 11 OR 12
7	Virtual Learning Platform*	14	3 AND 13

1. Pubmed

	Searching Term	Results
#1	students, nursing[MeSH Terms]	33,360
#2	("nursing"[MeSH Terms] OR "nursing"[All Fields] OR "nursings"[All Fields] OR "nursing"[MeSH Subheading] OR "nursing s"[All Fields]) AND "student*"[All Fields]	77,248
#3	#1 OR #2	77,248
#4	"virtual reality"[MeSH Terms]	8,195
#5	("virtual"[All Fields] OR "virtuality"[All Fields] OR "virtualization"[All Fields] OR "virtualized"[All Fields] OR "virtualizing"[All Fields] OR "virtuals"[All Fields]) AND "realit*"[All Fields]	26,509
#6	("virtual"[All Fields] OR "virtuality"[All Fields] OR "virtualization"[All Fields] OR "virtualized"[All Fields] OR "virtualizing"[All Fields] OR "virtuals"[All Fields]) AND "simulat*"[All Fields]	32,182
#7	("virtual"[All Fields] OR "virtuality"[All Fields] OR "virtualization"[All Fields] OR "virtualized"[All Fields] OR "virtualizing"[All Fields] OR "virtuals"[All Fields]) AND ("learning"[MeSH Terms] OR "learning"[All Fields] OR "learn"[All Fields] OR "learned"[All Fields] OR "learning s"[All Fields] OR "learnings"[All Fields] OR "learns"[All Fields]) AND "platform*"[All Fields]	2,146
#8	("head"[MeSH Terms] OR "head"[All Fields]) AND "Up"[All Fields] AND "display*"[All Fields]	2,294
#9	("head"[MeSH Terms] OR "head"[All Fields]) AND ("mount"[All Fields] OR "mounted"[All Fields] OR "mounts"[All Fields]) AND "display*"[All Fields]	2,381
#10	("virtual"[All Fields] OR "virtuality"[All Fields] OR "virtualization"[All Fields] OR "virtualized"[All Fields] OR "virtualizing"[All Fields] OR "virtuals"[All Fields]) AND "education*"[All Fields]	23,607
#11	("virtual"[All Fields] OR "virtuality"[All Fields] OR "virtualization"[All Fields] OR "virtualized"[All Fields] OR "virtualizing"[All Fields] OR "virtuals"[All Fields]) AND "patient*"[All Fields]	38,332
#12	("virtual"[All Fields] OR "virtuality"[All Fields] OR "virtualization"[All Fields] OR "virtualized"[All Fields] OR "virtualizing"[All Fields] OR "virtuals"[All Fields]) AND "game*"[All Fields]	3,972
#13	#4 OR #5 OR #6 OR #7 OR #8 OR #9 OR #10 OR #11 OR #12	87,973
#14	#3 AND #13	1,598

2. Embase

	Searching Term	Results
#1	nursing student'/exp OR 'nurse student' OR 'nursing students' OR 'student nurse' OR 'students, nursing' OR 'nursing student'	42828
#2	virtual reality'/exp OR 'virtual realit*'	41483
#3	virtual reality simulator'/exp OR 'virtual reality simulation devices' OR 'virtual reality simulation system' OR 'vr simulation system' OR 'vr simulator' OR 'virtual reality simulator' OR 'virtual simulat*'	4272
#4	virtual reality system'/exp OR 'medpresence' OR 'virtual reality interface' OR 'vr interface' OR 'vr system (virtual reality)' OR 'virtual reality system'	4250
#5	virtual reality head mounted display'/exp OR 'head-mounted virtual reality' OR 'hmd-vr' OR 'optivu' OR 'virtual reality glasses' OR 'virtual reality head set' OR 'virtual reality headset' OR 'virtual reality helmet' OR 'virtual reality hmd' OR 'vr glasses' OR 'vr head mounted display' OR 'vr head set' OR 'vr head-worn display' OR 'vr headset' OR 'vr helmet' OR 'vr-hmd' OR 'vrhmd' OR 'virtual reality head mounted display'	1916
#6	#2 OR #3 OR #4 OR #5	43512
#7	#1 AND #6	403

3. Cochrane

	Searching Term	Results
#1	MeSH descriptor: [Students, Nursing] explode all trees	931
#2	(Nursing Student*)	5167
#3	#1 OR #2	5167
#4	MeSH descriptor: [Virtual Reality] explode all trees	1304
#5	(virtual realit*)	8249
#6	(Virtual Simulat*)	2803
#7	(Virtual Learning Platform*)	401
#8	(Head Up Display*)	847
#9	(Head Mounted Display*)	516
#10	(virtual education*)	4678
#11	(virtual patient*)	25014
#12	(virtual game*)	1823
#13	#4 OR #5 OR #6 OR #7 OR #8 OR #9 OR #10 OR #11 OR #12	31367
#14	#3 AND #13	399

4. CINAHL

Searching Term	Results
((nursing student*) AND ((virtual realit*) OR (VR) OR (virtual reality simulat*) OR (virtual patient*) OR (virtual simulat*) OR (head mounted display) OR (virtual game*)))	827

5. ACM Digital Library

Searching Term	Results
((nursing student*) AND ((virtual realit*) OR (VR) OR (virtual reality simulat*) OR (virtual patient*) OR (virtual simulat*) OR (head mounted display) OR (virtual game*)))	196

https://doi.org/10.7586/jkbns.25.088
