# Supplementary material for: Effects of virtual reality-based simulation education for nursing students: a systematic review and meta-analysis
Source: J Korean Biol Nurs Sci. 2026 Feb 27;28(1):43–65. doi: 10.7586/jkbns.25.088 (PMC13268350; doi:10.7586/jkbns.25.088)
Supplement: Supplementary file 2 [file jkbns-25-088-Appendix-2.pdf]

JKBNS

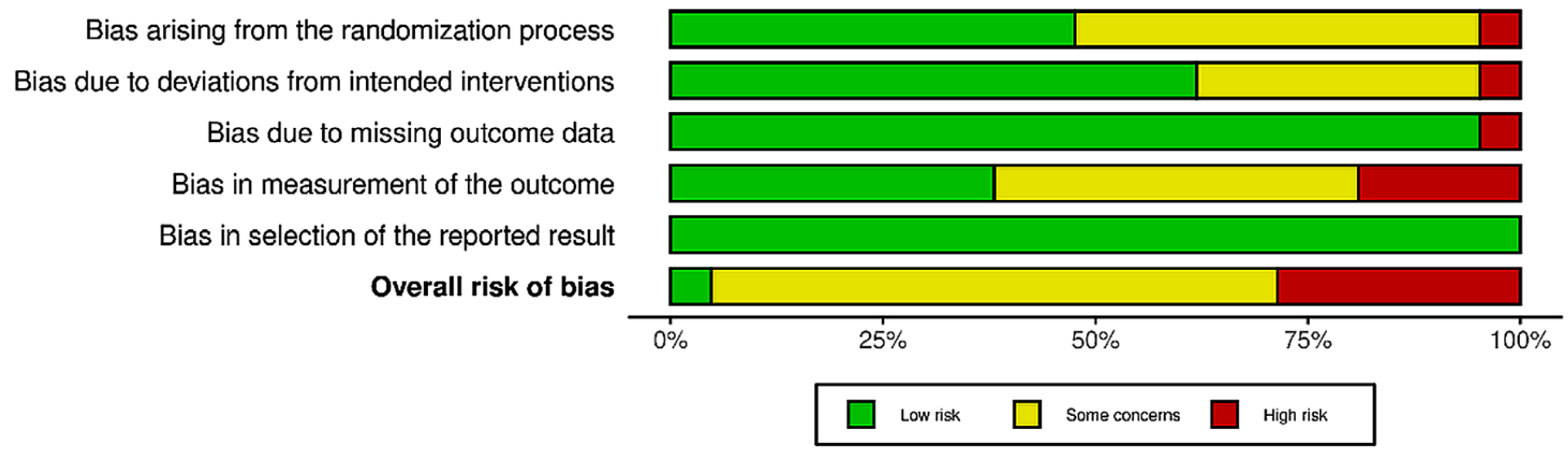

Appendix 2A. Risk of bias assessment

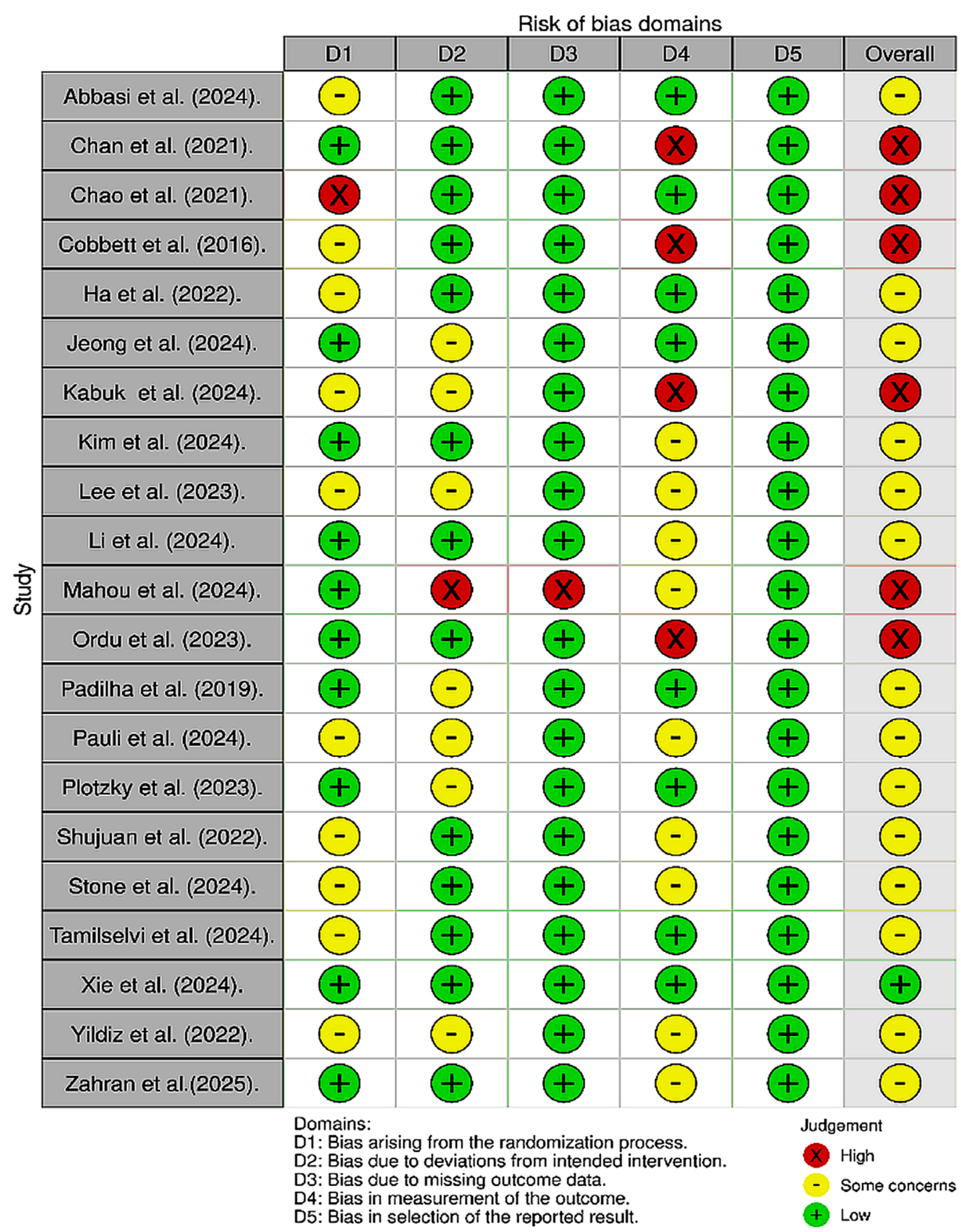

Appendix 2B. Risk of Bias Traffic-Light Plot
