# Supplementary material for: Effects of virtual reality-based simulation education for nursing students: a systematic review and meta-analysis
Source: J Korean Biol Nurs Sci. 2026 Feb 27;28(1):43–65. doi: 10.7586/jkbns.25.088 (PMC13268350; doi:10.7586/jkbns.25.088)
Supplement: Supplementary file 3 [file jkbns-25-088-Appendix-3.pdf]

Appendix 3. Studies included in Systematic Review and Meta-analysis

A1. Abbasi M, Asadoola Y, Ebrahimi H, Bahonar E, Dabirian Z, Esmaeili SM, et al. Comparison of mannequin-based simulation training method with virtual training method on nursing students' learning cardiopulmonary resuscitation: a controlled randomized parallel trial. Iranian Journal of Nursing and Midwifery Research. 2024;29(4):438–445. https://doi.org/10.4103/ijnmr.ijnmr_222_23

A2. Chan HY, Chang HC, Huang TW. Virtual reality teaching in chemotherapy administration: randomised controlled trial. Journal of Clinical Nursing. 2021;30(13–14):1874–1883. https://doi.org/10.1111/jocn.15701

A3. Chao YC, Hu SH, Chiu HY, Huang PH, Tsai HT, Chuang YH. The effects of an immersive 3d interactive video program on improving student nurses' nursing skill competence: a randomized controlled trial study. Nurse Education Today. 2021;103:104979. https://doi.org/10.1016/j.nedt.2021.104979

A4. Cobbett S, Snelgrove-Clarke E. Virtual versus face-to-face clinical simulation in relation to student knowledge, anxiety, and self-confidence in maternal-newborn nursing: a randomized controlled trial. Nurse Education Today. 2016;45:179–184. https://doi.org/10.1016/j.nedt.2016.08.004

A5. Ha YO, Kwon SJ, Kim J, Song JH. Effects of nursing skills practice using VR (virtual reality) on competency and confidence in nursing skills, learning self-efficacy, and satisfaction of nursing students. Journal of Industrial Convergence. 2022;20(4):47–55. https://doi.org/10.22678/JIC.2022.20.4.047

A6. Jeong S, Cha C. Effects of immersive virtual reality simulation–based maternity nursing education: a randomized controlled trial. Clinical Simulation in Nursing. 2024;97:101631. https://doi.org/10.1016/j.ecns.2024.101631

A7. Kabuk A, Taşcı SM, Türkoğlu İ, Göktaş S, Şendir M. Comparison of the effects of haptic virtual versus high-fidelity simulations on knowledge, skills and satisfaction of nursing students in cardiopulmonary resuscitation training. Clinical Simulation in Nursing. 2024;94:101586. https://doi.org/10.1016/j.ecns.2024.101586

A8. Kim J, Song JH, Ha YO. Effects of virtual reality cardiopulmonary resuscitation practice on the knowledge, skills, and attitudes of nursing students: a single-blind randomized controlled trial (RCT). Research in Community and Public Health Nursing. 2024;35(4):415-423. https://doi.org/10.12799/rcphn.2024.00689

A9. Lee JJ, Tsang VWY, Chan MMK, O'Connor S, Lokmic-Tomkins Z, Ye F, et al. Virtual reality simulation-enhanced blood transfusion education for undergraduate nursing students: a randomised controlled trial. Nurse Education Today. 2023;129:105903. https://doi.org/10.1016/j.nedt.2023.105903

A10. Li Y, Lv Y, Dorol RD, Wu J, Ma A, Liu Q, et al. Integrative virtual nursing simulation in teaching cardiopulmonary resuscitation: a blended learning approach. Australasian Emergency Care. 2024;27(1):37–41. https://doi.org/10.1016/j.auec.2023.07.006

A11. Mahou FZ, Decormeille G, Changuiti O, Mouhaoui M, Khattabi A. The effects of screen-based simulation on nursing students' acquisition of medication administration and dosage calculation skills: a randomized controlled trial. BMC Nursing. 2024;23(1):1–12. https://doi.org/10.5281/zenodo.17852038

A12. Ordu Y, Çalışkan N. The effects of virtual gaming simulation on nursing students' diagnosis, goal setting, and diagnosis prioritization: a randomized controlled trial. Nurse Education in Practice. 2023;68:103593. https://doi.org/10.1016/j.nepr.2023.103593

A13. Padilha JM, Machado PP, Ribeiro A, Ramos J, Costa P. Clinical virtual simulation in nursing education: randomized controlled trial. Journal of Medical Internet Research. 2019;21(3):e11529. https://doi.org/10.2196/11529

A14. Pauli V, Hughesdon K. Randomized controlled trial comparing simulation modalities in first year nursing students. Clinical Simulation in Nursing. 2024;89:101521. https://doi.org/10.1016/j.ecns.2024.101521

A15. Plotzky C, Loessl B, Kuhnert B, Friedrich N, Kugler C, König P, et al. My hands are running away–learning a complex nursing skill via virtual reality simulation: a randomised mixed methods study. BMC Nursing. 2023;22(1):222. https://doi.org/10.1186/s12912-023-01384-9

A16. Shujuan L, Mawpin T, Meichan C, Weijun X, Jing W, Biru L. The use of virtual reality to improve disaster preparedness among nursing students: a randomized study. Journal of Nursing Education. 2022;61(2):93–96. https://doi.org/10.3928/01484834-20211213-05

A17. Stone RT, Mgaedeh FZ, Pulley AN. Cognitive and physiological evaluation of virtual reality training in nursing. Ergonomics. 2024;67(11):1440–1452. https://doi.org/10.1080/00140139.2024.2337842

A18. Tamilselvi A, Pandurangan HP, Subashini SP. Effectiveness of virtual simulation on self-efficacy, learning and self-confidence among nursing students in north India: a randomised control trial. Nursing Journal of India. 2024;115(1):43–47

https://doi.org/10.48029/NJI.2024.CXV108

A19. Xie Z, Zou L, Wang Q, Chen Y, Li L, Liao Y, et al. Application of immersive virtual reality (IVR) for the care of common diseases of older adults course: a mixed methods study. Geriatric Nursing. 2024;58:399–409. https://doi.org/10.1016/j.gerinurse.2024.06.003

A20. Yildiz H, Demiray A. Virtual reality in nursing education 3D intravenous catheterization e-learning: a randomized controlled trial. Contemporary Nurse. 2022;58(2–3):125–137. https://doi.org/10.1080/10376178.2022.2051573

A21. Zahran H, Malak MZ, El-Qirem F, Asfour B. The effect of virtual reality airway management as a learning strategy on performance, self-efficacy, and emotional intelligence among nursing students in the West Bank/Palestine. Teaching and Learning in Nursing. 2025;20(1):e35–e42. https://doi.org/10.1016/j.teln.2024.07.023
