# Supplementary material for: Effects of virtual reality-based simulation education for nursing students: a systematic review and meta-analysis
Source: J Korean Biol Nurs Sci. 2026 Feb 27;28(1):43–65. doi: 10.7586/jkbns.25.088 (PMC13268350; doi:10.7586/jkbns.25.088)
Supplement: Supplementary file 4 [file jkbns-25-088-Appendix-4.pdf]

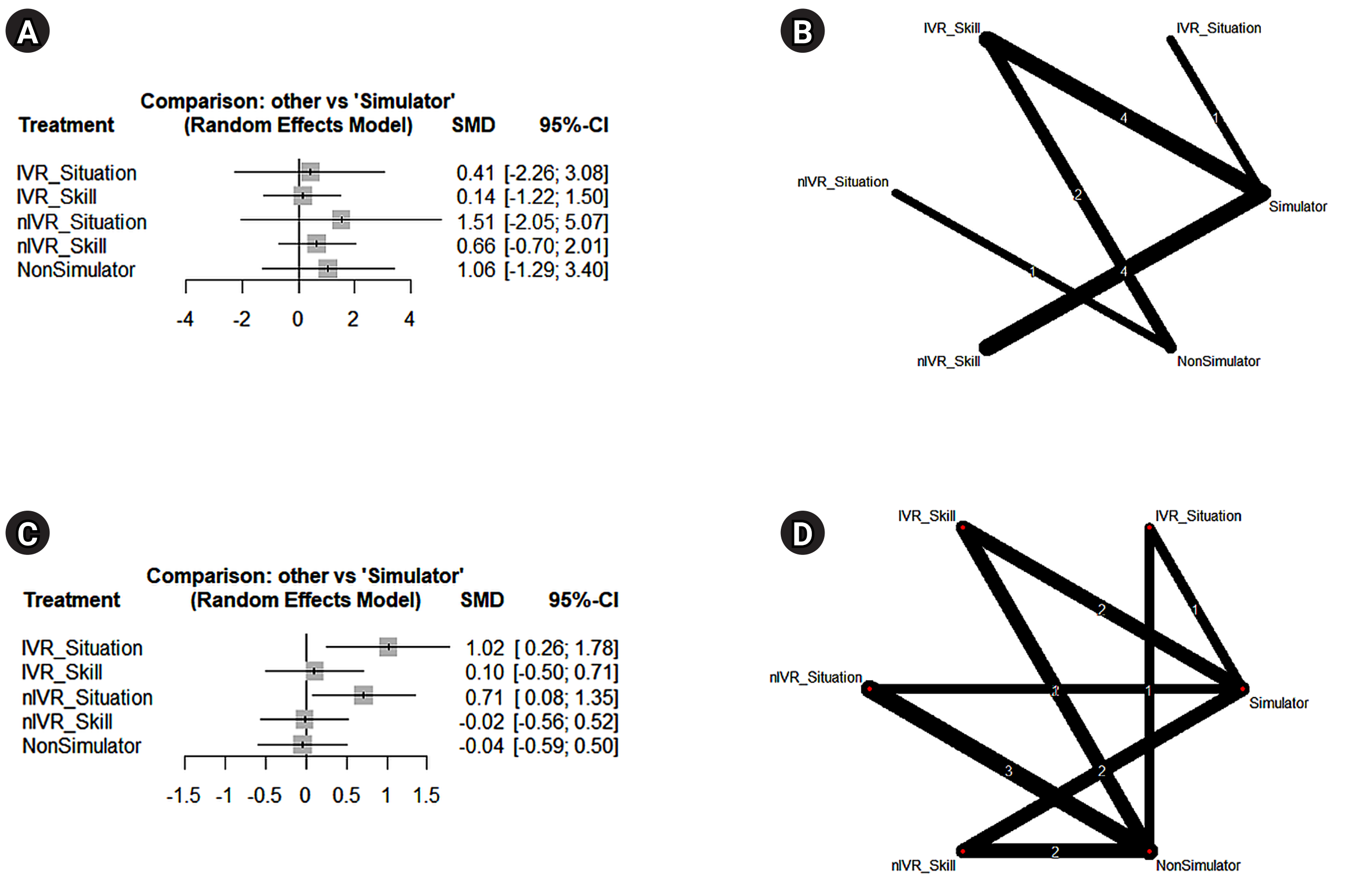

Appendix 4. Network meta-analysis results for key outcomes. (A) Network meta-analysis forest plot for nursing skill performance. (B) Network plot for nursing skill performance. (C) Network meta-analysis forest plot for knowledge. (D) Network plot for knowledge. SMD = Standardized mean difference; CI = Confidence interval; IVR = Immersive virtual reality; nIVR = non-Immersive virtual reality.
