# Supplementary material for: Effects of virtual reality-based simulation education for nursing students: a systematic review and meta-analysis
Source: J Korean Biol Nurs Sci. 2026 Feb 27;28(1):43–65. doi: 10.7586/jkbns.25.088 (PMC13268350; doi:10.7586/jkbns.25.088)
Supplement: Supplementary file 6 [file jkbns-25-088-Appendix-6.pdf]

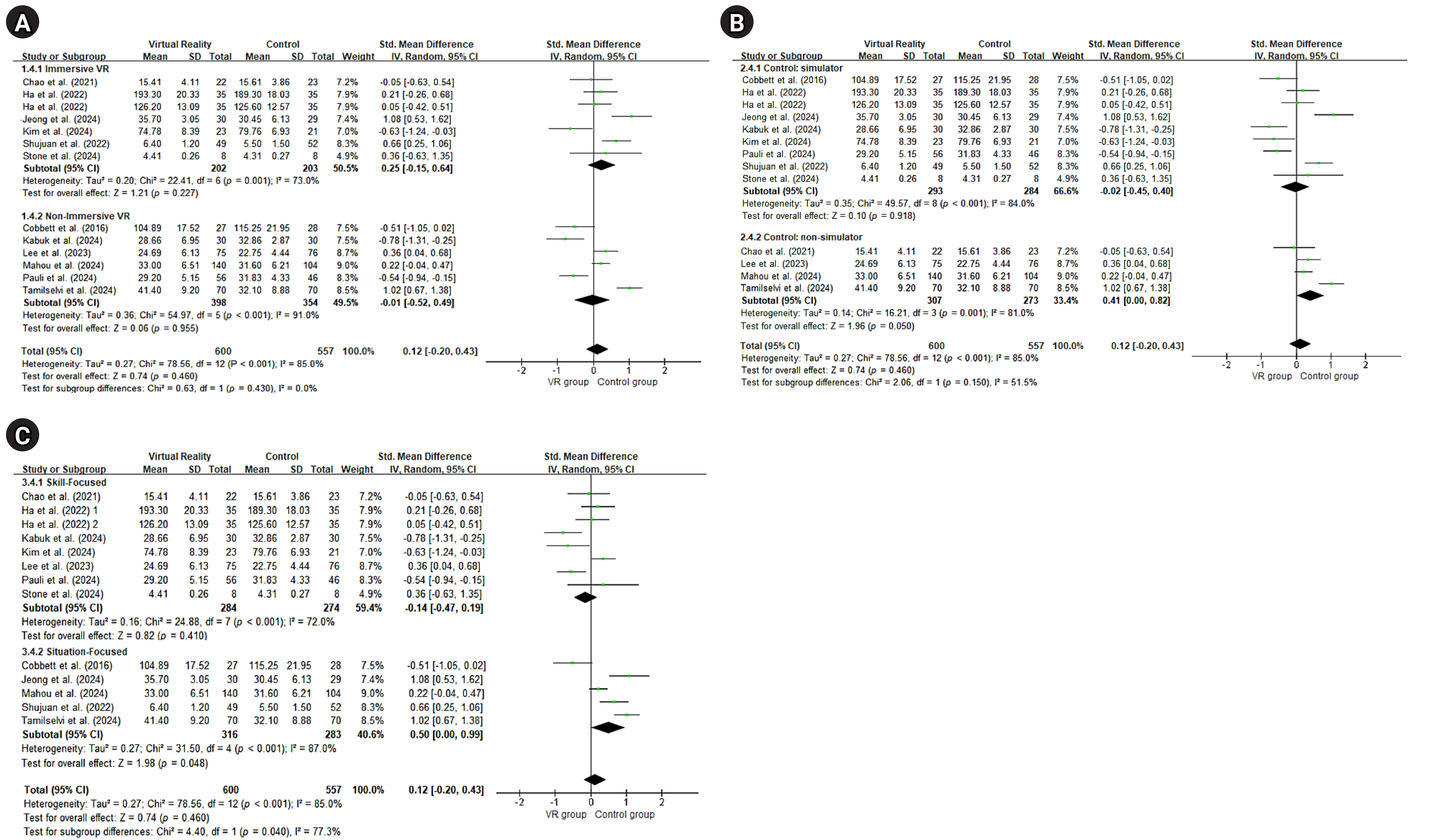

Appendix 6. Forest plot of the effect of virtual reality–based simulation on confidence in nursing performance. (A) Immersiveness of intervention (immersive vs non-immersive. (B) Control group (simulator vs non-simulator). (C) Contents type (skill focused vs situation focused). SD = Standard deviation; CI = Confidence interval; VR = Virtual reality.

https://doi.org/10.7586/jkbns.25.088
